# Draft Genome Sequence and Annotation of the Synthetic Textile Dye-Decolorizing Strain Bacillus amyloliquefaciens AD20, Isolated from a Dye Waste Pond

**DOI:** 10.1128/MRA.00645-21

**Published:** 2021-08-26

**Authors:** Imade Y. Nsa, Ayodeji A. Odunsi, Olukayode O. Amund

**Affiliations:** a Department of Microbiology, Faculty of Science, University of Lagos, Lagos, Nigeria; b Department of Biological Sciences, Faculty of Basic and Applied Sciences, Elizade University, Ilara-Mokin, Ondo State, Nigeria; University of Maryland School of Medicine

## Abstract

A limited number of Bacillus amyloliquefaciens genome sequences have been generated and are available in the public domain from soil, fermented foods, and plants. Here, we report the whole-genome sequence of *B. amyloliquefaciens* AD20, isolated from a dye pond with azo dye decolorization capabilities.

## ANNOUNCEMENT

Bacillus amyloliquefaciens, a member of the Bacillus subtilis group, is a Gram-positive rod which produces α-amylase. *B. amyloliquefaciens* is associated with fighting root pathogens (1) and the breakdown of synthetic textile dyes (2–5). *B. amyloliquefaciens* AD20, isolated from a dye waste pond (DWP) near a textile factory, has dye-reducing capabilities.

To isolate culturable bacteria from the DWP, 1 g of sediment was inoculated into 99 ml of sterile distilled water to make an initial 10^−2^ dilution and further series up to 10^−7^. Aliquots from 10^−4^ to 10^−6^ dilution tubes were inoculated onto tryptic soya agar (TSA) plates and incubated at 37°C for 24 h. After incubation, individual isolates selected from the mixed culture TSA plates were subcultured thrice on TSA to obtain pure cultures of the distinct bacterial isolates. Glycerol stocks of the isolates were stored at −70°C. *B. amyloliquefaciens* was one of the bacterial isolates from the screen that showed dye decolorization capabilities on a medium infused with the dyes.

To identify *B. amyloliquefaciens* AD20 by 16S rRNA gene sequencing (GenBank accession number MT365797) and for its whole-genome sequencing (WGS), genomic DNA was extracted according to the protocol for the ZR Quick-DNA fungal/bacterial miniprep kit (Zymo, USA) from approximately 20 mg of cells collected from an overnight culture on tryptic soya medium (37°C).

DNA quantification using a Qubit fluorometer and genome library preparation of the isolated DNA were conducted at FISABIO (Valencia, Spain). The DNA library preparation was performed using the Nextera XT library prep kit (FC-131-1024), and multiplexing was conducted using a Nextera XT index kit (FC-131-1096) (Illumina, USA). Sequencing of the paired-end reads was performed on an Illumina MiSeq instrument with 2 × 300-bp cycles.

The quality of the sequencing reads and draft genome were obtained using the QUAST version 5.0.2 evaluation tool (6, 7). In total, 5,467,278 paired-end raw reads were trimmed to 5,363,381 reads using FASTP version 0.20.1 (8), followed by assembly using Unicycler version 0.4.8.0 (9). The assembled contigs were annotated using the Rapid Annotations Subsystems Technology toolkit (RASTtk) version 1.073 (10, 11) on the Pathosystems Resource Integration Center (PATRIC) server version 3.6.9. Default parameters were used for all software.

The data obtained from RASTtk revealed 4,086 PATRIC protein-coding sequences, 3 rRNA genes, and 76 tRNA genes. The genome sequence comprised 4,003,399 bp in total, with an average short read coverage of 592.994×, and contained 28 contigs, with a GC content of 46.32%. The longest contig was 1,098,879 bp, and it had an *N*_50_ value of 1,016,292 bp. There were 3,339 proteins with assigned functions and 747 hypothetical proteins. The reference genome used for functional annotation with PATRIC was Bacillus amyloliquefaciens strain Fito_F321. Plasmid-related sequences and *Bacillus* toxin genes were absent. Using PATRIC version 3.6.9 (12), the National Database of Antibiotic Resistant Organisms (NDARO) (13), and the Comprehensive Antibiotic Resistance Database (CARD) version 3.1.3 (14), built into RASTtk, lincomycin and tetracycline antibiotic resistance genes were identified. Significantly, using PATRICbrc, genes were identified which code for the dye decolorization enzymes flavin mononucleotide (FMN)-dependent NADH azoreductase (EC 1.7.1.6) (PLF_1386_00006331), azoreductase (EC 1.7.1.6) (PLF_1386_00005892), and the spore coat laccase CotA (EC 1.10.3.2) (PLF_1386_00311525).

### Data availability.

The draft genome sequence of Bacillus amyloliquefaciens AD20 was deposited in PATRICbrc under the accession number 1390.742, and its project data are available under BioProject accession number PRJNA734188, BioSample accession number SAMN19487240, and SRA accession number SRR14875335.
